# Review of carcinogenicity of asbestos and proposal of approval standards of an occupational cancer caused by asbestos in Korea

**DOI:** 10.1186/s40557-015-0080-1

**Published:** 2015-12-30

**Authors:** Sanghyuk Im, Kan-woo Youn, Donghee Shin, Myeoung-jun Lee, Sang-Jun Choi

**Affiliations:** Wonjin Institute for Occupational & Environmental Health, Green Hospital, Seoul, South Korea; Catholic University of Daegu, Gyeongsan-si, South Korea

**Keywords:** Asbestos, Lung cancer, Job exposure matrix, Approval standard

## Abstract

Carcinogenicity of asbestos has been well established for decades and it has similar approval standards in most advanced countries based on a number of studies and international meetings. However, Korea has been lagging behind such international standards. In this study, we proposed the approval standards of an occupational cancer due to asbestos through intensive review on the Helsinki Criteria, post-Helsinki studies, job exposure matrix (JEM) based on the analysis of domestic reports and recognized occupational lung cancer cases in Korea. The main contents of proposed approval standards are as follows; ① In recognizing an asbestos-induced lung cancer, diagnosis of asbestosis should be based on CT. In addition, initial findings of asbestosis on CT should be considered. ② High Exposure industries and occupations to asbestos should be also taken into account in Korea ③ An expert’s determination is warranted in case of a worker who has been concurrently exposed to other carcinogens, even if the asbestos exposure duration is less than 10 years. ④ Determination of a larynx cancer due to asbestos exposure has the same approval standards with an asbestos-induced lung cancer. However, for an ovarian cancer, an expert’s judgment is necessary even if asbestosis, pleural plaque or pleural thickening and high concentration asbestos exposure are confirmed. ⑤ Cigarette smoking status or the extent should not affect determination of an occupational cancer caused by asbestos as smoking and asbestos have a synergistic effect in causing a lung cancer and they are involved in carcinogenesis in a complicated manner.

## Background

Carcinogenicity of asbestos has been well established for decades and it has similar approval standards of industrial accidents compensation in most advanced countries based on numerous studies and international meetings. However, Korea has been lagging behind such international standards. Approval standards of diseases due to asbestos in Korea have just followed Japanese standards of decades ago. They remained unchanged until 2013, without incorporating the latest asbestos studies. In 2013, new approval standards were proposed on enforcement decree of the Industrial Accident Compensation Insurance Act [1]. The new approval standards are as follows.

*Lung cancer, malignant mesothelioma, larynx cancer or ovarian cancer due to asbestos exposure, corresponding to any of the followings: 1) Accompanied by pleural thickening including pleural plaque or asbestosis; 2) Asbestos bodies or asbestos fibers found in sputum; and 3) Exposed to asbestos for 10 years or more (but cases with an exposure duration shorter than 10 years are also included if recognized as a disease caused by asbestos, based on consideration of the level of exposure, exposure duration and period between exposure and disease development)*

While the new standards cover a broader range of occupational cancers due to asbestos by including cancers at several sites as set out by the International Agency for Research on Cancer (IARC), the standards for the evidence of asbestos exposure are vague. So there are several challenging issues to determine an occupational cancer. First, whether to follow the International Labor Organization (ILO) classification or establish a separate standard for asbestosis diagnosis in the occupational cancer approval standards; Second, whether the presence of pleural plaque or pleural thickening alone qualifies as the evidence of asbestos exposure; Third, whether asbestos bodies or asbestos fibers found in sputum serve as the evidence of occupational asbestos exposure, and if they do, how many should be found; and finally in cases of asbestos exposure for 10 years or more, whether there is a difference between high concentration and low concentration exposure.

The first international expert meeting on ‘Asbestos, asbestosis, and cancer’ was convened in Helsinki in 1997 to discuss disorders in association with asbestos and to agree on the criteria for diagnosis and attribution with respect to asbestos [2, 3]. The group decided to name this document as the Helsinki Criteria. Subsequently, the Helsinki Criteria for asbestos-related lung cancers have been widely accepted and used for diagnosis and compensation in a number of countries including Germany, France, Finland and Australia.

These criteria have been highly controversial and an expert meeting in 2000 recommended making a radiographic diagnosis based on CT. Nevertheless, an intense debate is still ongoing for the occupational exposure standard of 25 fiber-years and histological standard. Several studies [4, 5] have reported an association between low concentration asbestos exposure and lung cancer, despite a rapid reduction in the asbestos use and exposure level with introduction of asbestos regulations in the 1980s. Against this backdrop, it is warranted to establish new approval standards of occupational cancers due to asbestos in Korea, based on international approval standards and the current research trend.

### Carcinogenicity of asbestos

The IARC concluded in 1977 and 1987 that asbestos qualifies as a human carcinogen [6, 7]. Since asbestos was listed in the First Annual Report on Carcinogens, evidence of carcinogenicity of asbestos has been reevaluated by the Institute of Medicine (IOM) of the National Academy of Sciences in 2006 [8] and by IARC in 2009 [9]. IARC concluded that exposure to all forms of asbestos is associated with an increased risk of lung cancer and mesothelioma. In addition, it concluded that there was sufficient evidence from epidemiological studies that asbestos also caused cancer of the larynx and ovary, as well as limited evidence that it caused cancer of the colorectum, pharynx, and stomach. In general, these conclusions were consistent with the IOM evaluation [9].

### Helsinki criteria and subsequent new trend

In the Helsinki Criteria [3] for occupational diseases associated with asbestos exposure, radiological findings of small opacities, grade 1/0, are usually regarded as an early stage of asbestosis for the purpose of screening. In terms of pleural disease, 80 ~ 90 % of the plaques that are radiologically well defined are attributable to occupational asbestos exposure. Low exposures (0.01 fibers/ml or less) from work-related, household, and natural sources may induce pleural plaques. For diffuse pleural thickening, higher exposure levels may be required. An occupational history of brief or low-level exposure should be considered sufficient for mesothelioma to be designated as occupationally related. A minimum of 10 years from the first exposure is required to attribute the mesothelioma to asbestos exposure, though in most cases the latency interval is longer. Smoking has no influence on the risk of mesothelioma. In the case of lung cancer, 1 year of heavy exposure (eg, manufacture of asbestos products, asbestos spraying, insulation work with asbestos materials, demolition of old buildings) or 5-10 years of moderate exposure (eg, construction, shipbuilding) may increase the lung cancer risk 2 fold or more. At least 10 years should have passed since the first asbestos exposure. A cumulative exposure of 25 fiber-years is estimated to increase the risk of lung cancer 2-fold. The presence of asbestosis is an indicator of high exposure. Asbestosis may also contribute some additional risk of lung cancer beyond that conferred by asbestos exposure alone. Heavy exposure, in the absence of radiologically diagnosed asbestosis, is sufficient to increase the risk of lung cancer. A 2-fold risk of lung cancer is related to retained fiber levels of 2 million amphibole fibers (>5 μm) per gram of dry lung tissue or 5 million amphibole fibers(>1 μm) per gram of dry lung tissue. This lung fiber concentration is approximately equal to 5000 to 15,000 asbestos bodies per gram of dry tissue, or 5 to 15 asbestos bodies per milliliter of bronchoalveolar lavage fluid. When asbestos body concentrations are less than 10,000 asbestos bodies per gram of dry tissue, electron microscopic fiber analyses are recommended.

### Use of CT in asbestos-related lung diseases

Several studies have announced the incidence of lung cancer is higher if there is no asbestosis on simple chest films. Wilkinson et al. found that after adjustments for gender, age, smoking history and area of referral, the odds ratio (OR) was 2.03 for 211 patients with a median ILO chest radiograph score of >1/0, whereas the OR was 1.56 in 738 patients with a score of <0/1 (95 % CI:1.02–2.39) [10].

The review pointed to a standardized mortality ratio (SMR) of 3.11 for lung cancer among Quebec miners and millers with small opacities in chest radiographs, a marker for asbestosis. However, the SMR was also elevated at 3.30 (95 % CI:2.32–4.62) in workers with radiographic abnormalities other than small opacities. Banks et al. point out that 11 out of the 37 in this category had a ‘large opacity’, not a feature of asbestosis, so that the SMR for lung cancer was apparently increased among those with radiological abnormalities other than asbestosis [11].

In a chest X-ray study on lung cancer in the Wittenoom cohort, Klerk et al demonstrated an increase in the relative risk (RR) with increasing cumulative exposure to asbestos, in the absence of radiographic asbestosis; the presence of asbestosis conferred an additional risk, but with a less steep slope for the dose-response line [12]. In a chest radiograph-based study of asbestos-cement workers in Ontario, Finkelstein found an increase in the RR in the absence of radiographic asbestosis [13].

High resolution computed tomography (HRCT) is already being used in many countries for diagnosis of lung diseases due to asbestos, due weaknesses of simple chest radiography, including a low diagnosis rate of asbestosis-related lung diseases and difficulty in early detection. Results from 2 studies of low dose CT use for lung cancer screening in workers with recent asbestos exposure support its usefulness, in particular, for screening of lung cancers [14]. CT is a diagnostic tool that is already being used in advanced countries. A recent study demonstrated remarkable usefulness of spiral CT in terms of sensitivity, specificity and positive predictive value in early diagnosis of lung cancers (Table 1) [15, 16].

**Table 1:** Detection rate, sensitivity, specificity and positive predictive value of computed tomography (CT) screening sturdies [17]

Study	Lung cancer +	Lung cancer -	Total	Results
Sone et al, 1998 [15], Sone, 2000 [18]				
Initial screening in 1996				Detection rate 0.4 %
CT +	25	305	330	Sensitivity 57 %
CT -	19	5965	5984	Specificity 95 %
Total	44	6270	6314	Predictive value 8 %
First annual repeat in 1997				Detection rate 0.6 %
CT +	28	169	197	Sensitivity 85 %
CT -	5	4823	4828	Specificity 97 %
Total	33	4992	5025	Predictive value 14 %
Second annual repeat in 1998				Detection rate 0.2 %
CT +	9	164	173	Sensitivity 100 %
CT -	0	4867	4867	Specificity 97 %
Total	9	5031	5040	Predictive value 5 %
Henschke et al, 1999 [19]				
Initial screening				Detection rate 2.7 %
CT +	27	206	233	Sensitivity 100 %
CT -	0	767	767	Specificity 79 %
Total	27	973	1000	Predictive value 12 %
First annual repeat				Detection rate 0.6 %
CT +	6	24	30	Sensitivity 100 %
CT -	0	970	970	Specificity 98 %
Total	6	994	1000	Predictive value 20 %
Vehmas et al, 2000 [20]				
Initial screening				Detection rate 0.8 %
CT +	5	60	65	Sensitivity 100 %
CT -	0	537	537	Specificity 90 %
Total	5	597	602	Predictive value 8 %

CT+ means the lung cancer was detected by CT

CT- means the lung cancer was not detected by CT

Lung Cancer + means the lung cancer was diagnosed by biopsy

Lung Cancer- means the lung cancer was not diagnosed by biopsy

#### Standards for asbestosis based on CT

For the diagnosis of cancer from asbestos, it is the evidence of exposure of asbestos to be diagnosed asbestosis or pleural thickening. Differentiating idiopathic pulmonary fibrosis from asbestosis is important because of legal and compensatory issues [21]. Asbestosis and idiopathic pulmonary fibrosis have similar histopathologic appearances and similar radiographic manifestations.

Akira et al. [22] studied 80 patients with asbestosis and 80 patients with idiopathic pulmonary fibrosis, using a large-scale cohort study of asbestos fiber workers in Sennan industrial area of Osaka region of Japan. Two chest radiologists who were unaware of the clinical and pathologic data, assessed the type and distribution of parenchymal and pleural abnormalities on high-resolution CT, and the final decisions on CT findings were reached by consensus. The results are as follows.

① A combination of subpleural dots and subpleural lines was found in 49 (61 %) of the 80 patients with asbestosis and in 10 (13 %) of the 80 patients with idiopathic pulmonary fibrosis.
② A combination of subpleural dots, subpleural lines, and parenchymal bands was found in 28 (35 %) of the 80 patients with asbestosis; however, this combination was found in only one (1 %) of the 80 patients with idiopathic pulmonary fibrosis.
③ A combination of subpleural dots, subpleural lines, parenchymal bands, and mosaic perfusion was found in 17 (21 %) of the 80 patients with asbestosis and in none of the 80 patients with idiopathic pulmonary fibrosis.
④ A combination of visible bronchioles, bronchiolectasis within consolidation, and honeycombing was found in 28 (35 %) of the 80 patients with idiopathic pulmonary fibrosis and in only two (3 %) of the 80 patients with asbestosis.
⑤ Parenchymal bands were found in three (21 %) of 14 patients with asbestosis without pleural disease and 35 (53 %) of 66 patients with asbestosis with pleural disease. Parenchymal bands were found in 33 (77 %) of 43 patients with diffuse pleural thickening.
⑥ Fibrotic consolidation was found in 26 (60 %) of 43 patients with diffuse pleural thickening. Parenchymal bands and fibrotic consolidation were significantly more common in patients with diffuse pleural thickening.
⑦ In patients with asbestosis without pleural disease, subpleural dots, subpleural lines, and mosaic perfusion were more common and bronchiolectasis within consolidation, visible intralobular bronchioles, and honeycombing were less common.
⑧ Pleural disease was found in 66 (83 %) of 80 patients with asbestosis. Forty-six patients with asbestosis had pleural plaques, and 43 patients with asbestosis had diffuse pleural thickening. Twenty-three patients with asbestosis had both pleural plaques and pleural thickening. Pleural disease was found in three (4 %) of the 80 patients with idiopathic pulmonary fibrosis. These three patients had diffuse pleural thickening and no pleural plaques. In these three patients, parenchymal bands were found.

Subpleural dotlike or branching opacities, subpleural curvilinear lines, mosaic perfusion and parenchymal bands were found in asbestosis patient with statistical significance (*p* < 0.001). Instead of dotlike opacities, visible intralobular bronchioles, bronchiolectasis within fibrotic consolidation and honeycombing were often found in patients with the idiopathic pulmonary fibrosis (*p* < 0.0001). Ground-glass opacities, interlobular septal thickening, fibrotic consolidation and emphysema were common in both diseases.

Kim JS [23] reported that subpleural dotlike opacities and subpleural curvilinear opacities were more common in patients with asbestosis at an early stage by HRCT. With gradual progression, intralobular interstitial thickening or intralobular lines and interlobular septal thickening were found in patients with asbestosis by HRCT. And parenchymal bands, honeycombing appearances, ground-glass opacity(GGO) and traction bronchiectasis were found in patients with asbestosis at an advanced stage. GGO was mostly seen with reticular opacities, traction bronchiectasis and honeycombing appearances but was rarely observed alone so that GGO in asbestosis may suggest subtle fibrosis below the resolution of CT.

#### Asbestos exposure concentration

From the time of the first anecdotal reports on the occurrence of lung cancer in patients with asbestosis, there has existed an assumption that the processes of asbestos-mediated fibrogenesis and carcinogenesis are closely interwoven, leading to the postulation that the fibrosis is an obligate causal precursor for the cancer. Based on such assumption, fibrosis was recognized as a necessary phase preceding cancer. In reviewing 1930s case reports on this association, Nordmann suggested that the lung cancer has its origins in the bronchiolo-alveolar hyperplasia that accompanies late stage asbestosis, as in other forms of diffuse interstitial fibrosis. In effect, the fibrosis-cancer hypothesis postulates that asbestos cannot induce lung cancer by itself, but only through an intermediary and obligatory step of interstitial fibrosis (asbestos → asbestosis → cancer) [24].

Several studies have announced that even if there is no asbestosis in the lungs on chest X-ray, the risk of lung cancer is increased. Therefore, if there is no asbestosis in the lungs, standards of Helsinki (25fibers × ml^-1^ × years) have been used as asbestos exposure certification standards in many countries. However, recent papers criticized that 25 fiber-years is too high. In the investigation of the South Carolina asbestos textile workers, Dement et al. [25] found a SMR of 2.59 and a standardized risk ratio of 2.63 for white males (95 % CI:1.20–5.75) at exposures as low as the range of 2.7–6.8 fiber-years. The estimated cumulative exposure of 2.7–6.8 fiber-years would be in the range for the reference group. These findings indicate that for this cohort an increase in the lung cancer rate occurred at cumulative exposures insufficient for induction of histological asbestosis, so that this observation constitutes a falsification factor for the fibrosis-cancer hypothesis.

Gustavsson et al. [5] demonstrated that the relative risk of lung cancer increased monotonically with cumulative dose of asbestos in a population-based case-referent study (1038 cases and 2359 referents). The point estimates indicated a dose response curve that did not follow an exponential pattern, which would correspond to a straight line. The risk at the high concentration was lower than what was predicted with an exponentiated model but was closer to a linear model. The relative risk (exp(beta)) for the transformed variable was 1.494 (95 percent confidence interval (CI): 1.193, 1.871) per unit of exposure. The relative risk at a cumulative dose of x fiber-years was 1.494ln(x + 1). At 4 fiber-years, the risk was 1.494ln(4 + 1) = 1.90 (95 percent CI: 1.32–2.74).

#### Relation between smoking and asbestos in the causation of lung cancer

Cigarette smoke and asbestos are considered by most authorities to have a synergistic effect for lung cancer induction, and both are complex carcinogens that can affect multiple steps in the multistage process of carcinogenesis. The composite effect may range from less than additive to supramultiplicative, but the effect among insulation workers and as derived from case-referent studies approximates a multiplicative model, which has been accepted by many authorities for about the last 30 years. In either a multiplicative or a submultiplicative model, the combined effect of cigarette smoke and asbestos involves an interactive effect whereby the joint effect is greater than the sum of the two separate effects [26].

At least four mechanisms have been proposed as potential explanations for the synergy between cigarette smoke and asbestos. (1) Cigarette smoke may facilitate penetration of asbestos fibers into bronchial walls [27]. (2) Carcinogens in cigarette smoke such as benzopyrene may be adsorbed onto asbestos fibers with subsequent delivery of the carcinogens into cells at high concentration [28]. (3) Cigarette smoke may interfere with the clearance of asbestos from the lungs. Churg and Stevens recorded elevated concentrations of asbestos fibers in the airway tissues of smokers in comparison to non-smokers, for both amosite (~6-fold) and chrysotile (~50-fold), especially for short fibers [29]. (4) Free fatty acids in cigarette smoke may translocate iron into cell membranes, with enhancement of cell sensitivity to oxidants such as active oxygen species [30].

#### Relation between larynx cancer and lung cancer

Committee on Asbestos [31] reported that there is a dose-response relationship between larynx cancer and asbestos exposure based on 9 large-scale cohort studies and meta analysis of cohort and case-control studies. It also noted that larynx cancer and lung cancer have the same pathogenesis and effect of smoking. As the larynx is anatomically equivalent to the lungs, asbestos-induced pathogenesis of larynx cancer is the same as that of lung cancer: the larynx provides a direct route of passage for asbestos fibers as the lungs; asbestos fibers are accumulated in the larynx in the same manner and cause inflammation or damage; the larynx consists of squamous cells as the lungs; and larynx cancer results from squamous metaplasia and dysplasia. In counties that recognize a larynx cancer as an occupational disease, its approval standards are the same as the criteria for a lung cancer.

### Approval standards in other countries

1. Approval standards in Japan (Tables 2, 3) [32].

**Table 2:** Approval standards

Disease	Requirements for recognition
Asbestosis (including complications of asbestosis)	A disease occurring in a worker exposed to asbestos, corresponding to either ① or ② according to the pneumoconiosis management classification (management 1 ~ 4) under the Pneumoconiosis Act. In addition, the occupational disease is judged after determination of the pneumoconiosis management classification by the prefectural labor minister, in principle. **·** Management 4 asbestosis (pneumoconiosis due to asbestosis) ② Complications of Management 2, Management 3, or Management 4 asbestosis^1^
Malignant mesothelioma	(1) Malignant mesothelioma in the pleura, peritoneum, pericardium or tunica vaginalis testis of a worker exposed to asbestos; it is recognized as an occupational disease in case the chest X-ray images (type 1~4) show asbestosis findings as specified in the Pneumoconiosis Act or the work period with asbestos exposure corresponds to either ① or ②. However, cases with less than 10 years since the first occupational asbestos exposure are excluded. ① Type 1 or higher asbestosis findings in chest X-ray images ②Engaged in work involving asbestos exposure for at least 1 year ※ As it is challenging to diagnose malignant mesothelioma, it is important to confirm malignant mesothelioma with pathology results but in case pathology results are not available, the case should be judged by comprehensively considering clinical test results, imaging findings, clinical course and differentiation with other diseases.
Lung cancer	'Primary lung cancer' in an worker exposed to asbestos; it is recognized as an occupational disease if corresponding to any of ① to ⑥. However, cases with less than 10 years since the first occupational asbestos exposure are excluded. **·** Presence of asbestosis findings^2^ ② Pleural plaque findings + engaged in work involving asbestos exposure for at least 10 years^3^ ③ Broad range of pleural plaque findings^4^ + engaged in work involving asbestos exposure for at least 1 year ④ Findings of asbestos bodies or asbestos fibers^5^ + engaged in work involving asbestos exposure for at least 1 year ⑤ Complication of diffuse pleural thickening ⑥ Engaged in 3 specific types of work^6^ + engaged in work involving asbestos exposure for at least 5 years^7^
Positive asbestos pleural fluid	As pleural fluid may be present with various causes (including tuberculous pleurisy and rheumatoid pleurisy) other than asbestos, diagnosis of positive asbestos pleural fluid should rule out the cause of pleural fluid other than asbestos. Since it may make its diagnosis highly challenging, recognition of an occupational disease is judged in discussion between the Labor Standards Inspection Office Director and the Ministry of Health, Labour and Welfare Main Office.
Diffuse pleural change	Diffuse pleural thickening present in a worker exposed to asbestos; the thickness should meet the following standards and be accompanied by apparent respiratory dysfunction. It is recognized as an occupational disease if the work period involving asbestos exposure is at least 3 months (meeting all of ①~③ as follows). ① Engaged in work involving asbestos exposure for at least 3 years ② Apparent respiratory dysfunction: Vital capacity (%VC) of < 60 % ③ Pleural thickening beyond a certain extent: On chest CT images ◆ Unilateral thickening: Involving at least 1/2 of the chest wall ◆ Bilateral thickening: Involving at least 1/4 of the chest wall

^1^Complications refer to the followings. Pulmonary tuberculosis, Tuberculous pleurisy, Secondary bronchitis, Secondary bronchiectasis, Secondary pneumothorax

^2^Type 1 or higher asbestosis on chest X-ray images as specified in the Pneumoconiosis Act

^3^In case of asbestos product manufacturing, the work period since 1996 is calculated as 1/2 of the actual work period

^4^Broad range of pleural plaque refers to the case that apparent opacities are recognized that can be judged as a pleural plaque on chest X-ray images, the opacities are confirmed as a pleural plaque on chest CT images, and the pleural plaque accounts for 1/4 of the chest wall on chest CT images

^5^One of the followings are required for findings of asbestos bodies or asbestos fibers

Asbestos bodies of at least 5,000 per 1 g of dry lung tissue

Asbestos bodies of at least 5 in 1 ml of bronchial alveolar lavage fluid

Asbestos fibers (>5μm) of at least 2 million per 1 g of dry lung tissue

Asbestos fibers (>1μm) of at least 5 million per 1 g of dry lung tissue

Presence of asbestos bodies or asbestos fibers on a lung tissue section

^6^ "3 specific types of work" refers to asbestos spun product manufacturing, asbestos cement product manufacturing, and asbestos fit-up work

^7^ "Work period" refers the period of working in 1 of the above 3 types of work or their total period. However, for the work period after 1996, the period is calculated as 1/2 of the actual work period

**Table 3:** Definition of work involving asbestos exposure in the standards for industrial accident compensation

(1) Extraction, taking out or crushing of asbestos-containing ores or rocks or other asbestos refining-related work in an asbestos mine or its attached facilities
(2) Containing or transporting of the asbestos material in a warehouse
(3) Asbestos product manufacturing
(4) Asbestos spray
(5) Covering for insulation or heat insulation using a heat-resistant asbestos product or its repair
(6) Asbestos product processing, such as cutting
(7) Repair or demolition of a building or its attached facilities in which an asbestos product is used as a clothing material or construction material
(8) Repair or demolition of a ship or car in which an asbestos product was used
(9) Handling of a mineral (such as talc) containing asbestos as an impurity

In addition, work involving asbestos dust exposure at a level equivalent to or higher than the above types of work or indirect exposure around the above types of work is also applicable
2. Approval standards in France [33].

Diseases covered by compensation are ① diseases specified as an occupational disease due to asbestos under social security-related legislation, ② diseases commonly recognized as being attributable to asbestos, ③ cases of exposure to asbestos inside the French territory for which the causality with asbestos exposure is recognized by the Commission d’evaluationdes circonstances de l’exposition a l’miante(CECEA). Cases that are actually recognized are mostly asbestosis, positive pleural lesion, primary lung cancer and malignant mesothelioma. The Table 4 is asbestos-related diseases set out in the occupational disease list.

**Table 4:** Asbestos-related diseases in France

Occupational disease No	Disease
030A	Asbestosis: lung fibrosis diagnosed with X-ray images, irrespective of respiratory function test findings
030B	Positive pleural disease (unilateral/bilateral pericardial plaque or pleural plaque with or without calcification confirmed with tomograms, pleuritis, diffuse or localized thickening of the pleura)
030C	Malignant bronchial lesion with a pulmonary parenchymal lesion or positive pleural disease
030D	Primary malignant mesothelioma in the pleura, peritoneum or pericardium
030E	Other primary pleural mass
030Bis	Primary lung cancer

Of above diseases, in case of malignant mesothelioma and pericardial plaque or pleural plaque, asbestos exposure is estimated according to the ‘list of diseases for which asbestos exposure is proven with confirmation’ so that diagnosis in itself may qualify for compensation. For other diseases, causality with asbestos exposure should be demonstrated, and it is the responsibility of the CECEA. CECEA should include ① 2 members with professional knowledge on the assessment of risks resulting from asbestos exposure, ② 2 industrial medicine specialist or experts with professional knowledge on respiratory disorders or pneumoconiosis, and they are nominated by the Management Committee that is in charge of basic rights in the Fonds d’Indemnisation des Victimes de l’Amiante(FIVA). The Table 5 presents the diagnosis and work-relatedness assessment standards for asbestos-related lung cancer, malignant mesothelioma and pleural plaque.

**Table 5:** Approval standards of asbestos-related diseases in France [34]

	Medical diagnosis standards	Asbestos dust exposure standards	Latent duration
Asbestosis	Diagnosis of lung fibrosis with specific radiographic characteristics, irrespective of changes in pulmonary function test findings	2 years (List of directly related jobs)	Liability period: Up to 35 years after the end of exposure
Malignant mesothelioma	Histology; if it is insufficient, clinical course and radiological diagnosis	Routine exposure without a minimum period	Up to 40 years after the end of exposure
Lung cancer	Histology; if it is insufficient, clinical course and radiological diagnosis	10 year exposure + limited job group (work directly related to an asbestos-containing material(ACM), insulation using ACM, removal of an asbestos-containing insulation material, repair of a building in which asbestos is used, cutting and grinding of a material containing asbestos, shipbuilding and ship repair, manufacturing of an asbestos-containing friction material, maintenance performed with an asbestos-containing equipment)	Up to 40 years after the end of exposure
Pleural plaque	Calcification or pleural plaque in the pericardium or pleura, confirmed with CT	Routine exposure without a minimum period	Up to 40 years after the end of exposure

3) Approval standards in Germany

The Table 6 presents the diagnosis and work-relatedness assessment standards for asbestos-related lung cancer, malignant mesothelioma and pleural plaque in Germany.

**Table 6:** Approval standards of asbestos-related diseases in Germany [34]

	Medical diagnosis standards	Asbestos dust exposure standards	Latent duration
Asbestosis	Lung fibrosis validated with X-ray (ILO standards) or CT/HRCT	Several years	At least 10 years
Malignant mesothelioma	Proven diagnosis (histopathology and radiography, CT is preferred)	Even low level exposure is recognized	At least 10 years, in general
Lung cancer	Asbestosis-related lung cancer (even histologically mild asbestosis is sufficient)	Exposed to 25 fibers/ml-year	At least 10 years
	Major changes in the pleura due to asbestos		
Pleural plaque	Diagnosis with radiography, CT or histopathology	Even low level exposure is recognized	-

### Exposure status in Korea

In order to evaluate the exposure status of asbestos, we developed a General Population based Korean Job-Exposure Matrix (JEM) using domestic quantitative datasets on the exposure to asbestos. Available data were obtained from previous exposure-related study reports and the work environment monitoring data under Article 42 of the Industrial Safety and Health Act. Domestic literature mostly focused on the primary asbestos exposure group between 1984 and 1996 and therefore, it is possible to construct the JEM for 1984 ~ 1996 by using these reports. In case of the work environment monitoring data, the Korea Occupational Safety and Health Agency (KOSHA) database (DB) was established in 2002. However, as there is no data prior to 2002, this study used analysis data for 1995 ~ 2006 obtained from Seoul National University Graduate School of Public Health(SNU GSPH), an institution that has been analyzing most airborne asbestos samples collected during work environment monitoring. KOSHA DB was used for the work environment monitoring data of 2005 ~ 2008.

To build the JEM, exposure groups in collected data were reclassified by standardized industry and occupation codes. For industry codes, the 9th Revised Korean Standard Industrial Classification (KSIC) was used in order to reflect industrial characteristics of Korea as well as to ensure international comparability. For occupation codes, the 6th Korean Standard Classification of Occupations (KSCO) was used to reflect the International Standard Classification of Occupations (ISCO-08) finalized at the end of 2007. Two trained industrial hygienists classified exposure groups from collected data according to standard industry and occupation codes.

1. According to the established JEM, 88 industries and 75 occupations involved the exposure to asbestos (Tables 7, 8). By period, the highest exposure occurred in ‘knitting and weaving machine operators’ working at ‘manufacture of asbestos, mineral wools and other similar products’ with arithmetic mean concentration of 7.48 f/m*ℓ* in the 1980s, ‘wood and paper related machine operators’ of ‘manufacture of other articles of paper and paperboard not elsewhere classified’ with 3.5 f/m*ℓ* in the 1990s and ‘detergents production machine operators’ of ‘manufacture of surface-active agents’ with 2.45 f/m*ℓ* in the 2000s. Detailed information of JEM will be scheduled to be described in another article.

**Table 7:** Asbestos exposure levels by industries in Korea

Industry^a^	<1990	1991 ~ 1999	2000 ~ 2008	Total
Foamed Plastic Products		5.12		5.12
Other Articles of Paper and Paperboard n.e.c.^b^		3.54		3.54
Surface-Active Agents			2.45	2.45
Asbestos, Mineral Wools and Other Similar Products	7.48	0.91	0.02	2.04
Cast of Iron and Steel	1.54			1.54
Weaving of Man-Made Fiber Fabrics		1.52		1.52
Moulding Patterns, Moulds and Industrial Patterns			1.51	1.51
Sale of Motor Vehicle New Parts and Accessories	1.41			1.41
Cutting, Shaping and Finishing of Stone			1.18	1.18
Paperboard Boxes and Containers			0.98	0.98
Industrial Un-vulcanized Rubber Products			0.96	0.96
Other Paper and Paperboard		0.00	1.61	0.81
Spinning of Wool			0.74	0.74
Repair Services of Motor Vehicles Specializing in Parts	0.93	0.56		0.68
Tires and Tubes			0.66	0.66
Synthetic Resin and Other Plastic Materials		0.04	0.83	0.63
Stone Products for Construction	0.46	0.74		0.60
Abrasive Articles		0.56		0.56
Taps, Valves and Similar Products			0.56	0.56
Other Parts and Accessories for Motor Vehicles n. e. c.			0.54	0.54
Synthetic Rubber			0.47	0.47
General Repair Services of Motor Vehicles			0.44	0.44
Other Parts and Accessories for Motor Vehicles n. e. c.	0.42			0.42
Other Insulated Wire and Cable			0.36	0.36
General Paints and Similar Products			0.32	0.32
Other Maintenance and Repair Services of General Machinery	0.23			0.23
Other Structural Metal Products			0.21	0.21
Electric Lamps and Electric Bulbs			0.20	0.20
Sections for Ships		0.06	0.24	0.18
General Construction		0.17		0.17
Insulated Codes Sets and Other Conductors for Electricity			0.12	0.12
Research and Experimental Development On Other Engineering			0.12	0.12
Sanitary Paper Products			0.12	0.12
Other Special Purpose Machinery, n.e.c.			0.11	0.11
Synthetic Rubber and of Plastics in Primary Forms			0.11	0.11
Paper Sacks and Paper Bags			0.11	0.11
Rubber Products			0.11	0.11
Aircraft Parts and Accessories		0.095		0.095
Parts and Accessories for Motor Vehicles and Engines		0.183	0.001	0.092
Building of Steel Ships		0.076		0.076
Special Yarns and Tire Cord Fabrics		0.073		0.073
Other Unclassified Non-metallic Minerals n. e. c.		0.069		0.069
Other Refractory Ceramic Products		0.064		0.064
Adhesives and Gelatin			0.055	0.055
Hot Rolled, Drawn and Extruded Iron or Steel Products		0.040		0.040
Apartment Building Construction		0.039		0.039
Parts and Accessories for Motor Engines		0.073	0.002	0.038
Heat Treatment of Metals			0.034	0.034
Broadcasting and Wireless Telecommunication Apparatuses		0.028		0.028
Other Footwear			0.026	0.026
Agricultural and Forestry Machinery		0.046	0.003	0.024
Other Sound Equipment		0.022		0.022
General Electric Lighting Fixture			0.020	0.020
Pharmaceutical Goods Other Than Medicaments			0.016	0.016
Waste Treatment Services			0.016	0.016
Electric Motors and Generators		0.014		0.014
Supporting, Railway Transport Activities			0.014	0.014
Cellulose Fiber Cement Products			0.013	0.013
Disposal of Hazardous Waste			0.013	0.013
Other Plastic Products n.e.c.		0.012		0.012
Other Rubber Products n.e.c.		0.012		0.012
Passenger Motor Vehicles		0.023	0.000	0.012
Other Fertilizers and Nitrogen Compounds			0.012	0.012
Other Work trucks, Lifting and Handling Equipment		0.009		0.009
Saws, Saw Blades and Interchangeable Tools		0.009		0.009
Other Basic Iron and Steel		0.008		0.008
Machinery for Food, Beverage and Tobacco Processing		0.008		0.008
Forging of Metal			0.008	0.008
Packaging Plastics and Shipping Containers		0.008		0.008
All Other Chemical Products n.e.c.			0.007	0.007
Metal Pressed and Stamped Products		0.007		0.007
All Other Glass and its Products n.e.c.			0.007	0.007
Pottery and Ceramic Household or Ornamental Ware			0.006	0.006
Engraving, Cutting and Similar Processing of Metals or Other Materials			0.006	0.006
Other Electronic Valves, Tubes and Electronic Components n.e.c.		0.011	0.002	0.006
Pulp			0.006	0.006
Broadcasting via Cable, Satellite and Other Broadcasting		0.005		0.005
Hazardous Waste Collection			0.005	0.005
Other Domestic Electric Appliances		0.005		0.005
Other Electric Motors, Generators and Transformers			0.004	0.004
General Hospitals			0.004	0.004
Electric Power Generation			0.004	0.004
Powder Metallurgic Products			0.003	0.003
Basic Organic Petrochemicals		0.010	0.000	0.003
Pumps and Compressors			0.003	0.003
Industrial Process Control Equipment			0.002	0.002
Residential Property Management			0.002	0.002
Other Manufacturing n.e.c.			0.001	0.001
Total	1.78	0.41	0.25	0.39

^a^the 9th Korean Standard Industrial Classification code name

^b^not elsewhere classified

All data were presented arithmetic mean (f//m*ℓ*)

**Table 8:** Asbestos exposure levels by occupations in Korea

Occupation^a^	<1990	1991 ~ 1999	2000 ~ 2008	Total
Wood and Paper Related Machine Operators n.e.c.^b^		3.54		3.54
Knitting and Weaving Machine Operators	7.48	1.34		3.39
Detergents Production Machine Operators			2.45	2.45
Paper Products Production Machine Operators			1.61	1.61
Metal Casting Machine Operators	1.54			1.54
Weaving Machine Operators		1.52		1.52
Store Salespersons n.e.c.	1.41			1.41
Construction Stonemason			1.18	1.18
Plastic Products Production Machine Operators n.e.c.		1.72	0.06	1.06
Automobile Paint Mechanics			0.96	0.96
Tire and Rubber Products Production Machine Operators n.e.c.			0.96	0.96
Painting Machine Operators n.e.c.			0.78	0.78
Tire Production Machine Operators			0.66	0.66
Mineral Ore and Stone Products Processing Machine Operators	0.46	0.74		0.60
Brightener Production Machine Operators		0.56		0.56
Machine Tool Operators			0.56	0.56
Automobile Mechanics	0.93	0.56	0.00	0.51
Die and Mold Makers		0.01	0.75	0.51
Paper Box and Envelope Products Processing Machine Operators			0.45	0.45
Textile Processing Machine Operators		0.07	0.74	0.41
Chemical Material Grinding and Mixing Machine Operators			0.35	0.35
Chemical Material Distiller and Reactor Operators			0.35	0.35
Rubber Products Production Machine Operators		0.01	0.47	0.24
Automobile Parts Assemblers n.e.c	0.42	0.18	0.03	0.21
Metal Product Painting Machine Operators			0.21	0.21
Audio-Visual Equipment Assemblers		0.02	0.36	0.19
Elementary Workers in Construction		0.17		0.17
Ship Assemblers	0.23	0.04	0.24	0.16
Ship Mechanics		0.13		0.13
Engineering Research Managers			0.12	0.12
Sanitary Paper Products Processing Machine Operators			0.12	0.12
Industry Machinery Assemblers			0.11	0.11
Electrical Products Production Equipment Operators		0.01	0.20	0.10
Aircraft Assemblers		0.09		0.09
Nonmetal Products Related Production Machine Operators n.e.c.		0.07		0.07
Chemical Material Processing Machine Operators			0.06	0.06
Electrical Equipment Assemblers		0.01	0.07	0.05
Brick and Tile Moulding Machine Operators		0.05		0.05
Agricultural Machinery Assemblers		0.05		0.05
Construction Related Technical Worker		0.04		0.04
Automobile Engine Assemblers		0.07	0.00	0.04
Railroad Train Mechanics			0.04	0.04
Metal Heat Treatment Furnace Operators			0.03	0.03
Electrical, Electronic Parts and Products Assembler n.e.c.		0.03		0.03
Textile and Leather Related Workers			0.03	0.03
Rolling Mill Operators		0.02		0.02
Cement and Mineral Products Production Machine Operators			0.02	0.02
Pharmaceutical Products Production Machine Operators			0.02	0.02
Recycling Machine and Incinerator Operators			0.02	0.02
Cement and Lime Production Related Machine Operators			0.01	0.01
Recycling Machine and Incinerator Operator n.e.c			0.01	0.01
Automobile Assemblers		0.02	0.00	0.01
Construction Carpenters			0.01	0.01
General Machinery Assemblers		0.009		0.009
Ore and Metal Furnace Operators		0.008		0.008
Food Processing Related Machine Operating Occupations		0.008		0.008
Plastic Catapulting Machine Operators		0.008		0.008
Pottery and Porcelain Products Production Machine Operators			0.006	0.006
Electronic Parts Production Equipment Operators		0.011	0.002	0.006
Paper Pulp Plant Operators			0.006	0.006
Chemical Products Production Machine Operators n.e.c.		0.010	0.004	0.006
Telecommunication and Broadcast Transmission Equipment Technicians		0.005		0.005
Elementary Workers in Construction			0.005	0.005
Aircompressor Operators			0.005	0.005
Paper Processing Machine Operators		0.005		0.005
Forge Hammersmiths and Forging Press Workers			0.004	0.004
Electrical Parts Production Equipment Operators			0.004	0.004
Power Generation and Distribution Equipment Operators			0.004	0.004
Railway Transport Clerks			0.003	0.003
Glass Production and Processing Machine Operators n.e.c.			0.003	0.003
Metal Processing Machine Operators n.e.c.			0.003	0.003
Health, Social Welfare and Religion Related Occupations			0.003	0.003
Cooling and Heating System Operators			0.002	0.002
Lathe Machine Operators			0.002	0.002
Railroad Train and Electric Train Mechanics			0.002	0.002
Total	1.78	0.41	0.25	0.39

^a^the 9th Korean Standard Industrial Classification code name

^b^not elsewhere classified

All data were presented arithmetic mean (f//m*ℓ*)

#### Cases in Korea

Analyzed cases in Korea included 179 cases of lung cancer from the epidemiological survey between 1994 and 2011 by KOSHA, and 31 cases of lung cancer from the Occupational Lung Diseases Institute, after excluding 11 cases of 2012 and 9 cases of malignant mesothelioma confirmed between 2004 and 2011, from 51 cases between 2004 and 2012.

For KOSHA cases, the study by Ahn YS was used, and for the Occupational Lung Diseases Institute cases, the same methodology was applied and data from 2 sources were pooled for statistical analysis.

The incidence of occupational lung cancer in Korea was 0.11 per 100,000 and it was 0.06 for lung cancer due to asbestos. The occupational lung cancer incidence is increasing every year, as the case with the lung cancer due to asbestos. Lung cancer due to asbestos represents approximately 60 % of the entire occupational lung cancer cases (Table 9).

**Table 9:** Recognition per 100,000 workers insured of lung cancer in Korea

Recognized year	Insured population	Lung cancer case	Asbestos-related lung cancer case	Proportion of Asbestos related lung cancer	Lung cancer incidence rate	Asbestos-related lung cancer incidence rate
	A	B	C	C/B*100	B/A*10^6^	C/A*10^6^
1994	7,273,132	1	1	100	0.01	0.01
1995	7,893,727	2	1	50.0	0.03	0.01
1996	8,156,894	5	1	20.0	0.06	0.01
1997	8,236,641	0	0		0	0
1998	7,582,479	2	0	0.0	0.03	0
1999	7,441,160	5	3	60.0	0.07	0.04
2000	9,485,557	9	3	33.3	0.09	0.03
2001	10,581,186	8	6	75.0	0.08	0.06
2002	10,571,279	11	4	36.3	0.10	0.04
2003	10,599,345	16	9	56.2	0.15	0.08
2004	10,473,091	16	9	56.2	0.15	0.09
2005	11,059,194	14	6	42.8	0.13	0.05
2006	11,688,800	11	4	36.3	0.09	0.03
2007	12,528,884	22	15	68.2	0.18	0.12
2008	13,489,990	21	13	62.0	0.16	0.10
2009	13,884,929	12	7	58.3	0.09	0.05
2010	14,198,757	29	17	58.6	0.20	0.12
2011	14,362,378	26	18	69.2	0.18	0.13

From the Table 10, men accounted for 95 % of occupational lung cancer patients and the mean age at diagnosis was 53 ~ 55 years. Given that those aged 60 ~ 65 years represent for the highest proportion of asbestos-induced lung cancer patients in Japan, this age range is relatively young. It is speculated to be due to the tendency that workers diagnosed with lung cancer after retirement did not apply for an industrial accident, rather than indicating early detection of lung cancer. Of all lung cancer patients, smokers accounted for 56.7 %. By histology, adenocarcinoma was the most frequent, followed by squamous cell cancer and small cell cancer.

**Table 10:** General characteristics of the study subjects

Variables			No	Percent
Diagnostic year	1993-1995		9	4.3
	1996-2000		29	13.8
	2001-2005		61	29.0
	2006-2011		111	52.9
Gender	Men		199	94.8
	Women		11	5.2
Age at diagnosis (years)	30-39		7	3.3
	40-49		56	26.7
	50-59		102	48.6
	60-69		38	18.1
	70-79		7	3.3
	Mean ± S.D.^a^		53.5 ± 8.2	
	Mean ± S.D.^b^		55.4 ± 9.1	
Smoking status	Current-smoker		93	44.3
	Ex-smoker		26	12.4
	Non-smoker		71	33.8
	Unknown-		20	9.5
	Mean pack-years of current and ex-smoker^a^		18.2 ± 8.6	
	Mean pack-years of current and ex-smoker^b^		21.5 ± 13.4	
Pathologic findings	Non-small cell ca	Adenocarcinoma	77	36.7
		Squamous cell ca	50	23.8
		Large cell ca	1	0.5
		Unclassified	14	6.7
		subtotal	142	67.6
	Small cell ca		21	10
	Unknown		47	22.4

^a^Values are given as mean ± S.D. of lung cancer-asbestos exposure case report data in KOSHA; S.D. is the abbreviation of standard deviation

^b^Values are given as mean ± S.D. of lung cancer-asbestos exposure case report data in occupational lung diseases institute (2004-2011)

In terms of exposure characteristics of cases (Table 11), asbestos was a key carcinogen, accounting for 50 % of causative carcinogens, and the exposure duration was approximately 20 years. The latent duration was 23 ~ 26 years, indicating that lung cancer is diagnosed approximately 3 ~ 6 years after the end of exposure. Eighty-seven lung cancer cases were due to exposure to a single carcinogen and 92 cases involved exposure to multiple carcinogens. In case of lung cancer due to asbestos, the exposure duration was approximately 20 years, the latent duration was about 24 years, smokers accounted for approximately 60 %, and adenocarcinoma was the most frequent histology for KOSHA cases (Table 12). For Occupational Lung Diseases Institute data, the exposure duration was approximately 23 years, the latent duration was about 27 years, smokers accounted for 77 %, and adenocarcinoma was the most frequent histology. Eleven cases were due to exposure to a single carcinogen and 20 cases involved exposure to multiple carcinogens (Table 13).

**Table 11:** Exposure characteristics of the study subjects

Variables		No	Percent
Major carcinogen caused lung cancer	Asbestos	105	50
	Crystaline silica	42	20
	Cr6+	26	12.4
	Welding fume including Cr6+,nickel	18	8.6
	DEE^a^	5	2.4
	Rubber dust	2	1.0
	CTPV^b^ & PAH^c^	3	1.4
	Coke oven emission (COE)	1	0.5
Year at first exposure	-1969	12	5.7
	1970-1979	73	34.8
	1980-1989	91	43.3
	1990-1999	33	15.7
	2000-	1	0.5
Exposure duration(years)	<10	12	5.7
	≥10 and < 20	84	40
	≥20 and < 30	92	43.8
	≥30	22	10.5
	Mean ± S.D.^d^	19.8 ± 9.9	
	Mean ± S.D.^e^	22.5 ± 8.4	
Latent duration(years)	<10	5	2.4
	≥10 and < 20	62	29.5
	≥20 and < 30	95	45.2
	≥30	48	22.9
	Mean ± S.D.^d^	23.0 ± 9.9	
	Mean ± S.D.^e^	26.6 ± 7.5	

^a^DEE is the abbreviation of diesel engine exhaust; ^b^CTPV is coal tar pitch volatile; ^c^PAH is polyaromatic hydrocarbons

^d^Values are given as mean ± S.D. of lung cancer-asbestos exposure case report data in KOSHA; S.D. is the abbreviation of standard deviation

^e^Values are given as mean ± S.D. of lung cancer-asbestos exposure case report data in occupational lung diseases institute (2004-2011)

**Table 12:** The durations of exposure and latency, smoking status and pathologic types of compensated lung cancers by the kinds of main carcinogens in KOSHA, Korea (1994-2011)

Exposed substances (No. of lung cancer)		Exposure duration (years)	Latent duration (years)	Smoking status	No. (%)	Type of pathology	No. (%)
Asbestos (87)	Mean ± S.D.^a^	20.1 ± 7.3	24.0 ± 7.7	Current-smoker	37(47.4)	Adenocarcinoma	34(57.6)
	Median	21.0	24.0	Ex-smoker	10(12.8)	Squamous cell ca	15(25.4)
	Min^b^-Max^c^	2.5-38	7-40	Never smoker	31(39.7)	Others	10(17.0)
Crystalline silica (42)	Mean ± S.D.^a^	19.1 ± 7.0	24.4 ± 10.8	Current-smoker	13(37.1)	Adenocarcinoma	12(48.0)
	Median	18.3	21.5	Ex-smoker	6(17.1)	Squamous cell ca	10(40.0)
	Min^b^-Max^c^	7-32	7-61	Never smoker	16(45.7)	Others	3(12.0)
Cr6+ & welding fume (40)	Mean ± S.D.^a^	20.2 ± 7.2	21.5 ± 7.2	Current-smoker	20(54.1)	Adenocarcinoma	12(44.4)
	Median	20.0	21	Ex-smoker	3(8.1)	Squamous cell ca	11(40.7)
	Min^b^-Max^c^	9-41	9-41	Never smoker	14(37.8)	Others	4(14.8)
Others (10)	Mean ± S.D.^a^	18.1 ± 4.7	18.6 ± 4.5	Current-smoker	4(40.0)	Adenocarcinoma	6(66.7)
	Median	16.5	17.0	Ex-smoker	2(20.0)	Squamous cell ca	2(22.2)
	Min^b^-Max^c^	15-16	15-16	Never smoker	4(40.0)	Others	1(11.1)
Single exposure (87)	Mean ± S.D.^a^	19.6 ± 6.7	22.8 ± 8.0	Current-smoker	32(43.8)	Adenocarcinoma	22(46.8)
	Median	20.0	22.0	Ex-smoker	6(8.2)	Squamous cell ca	16(34.0)
	Min^b^-Max^c^	2.5-35.0	7-41.0	Never smoker	35(47.9)	Others	9(12.3)
Co-exposure (92)	Mean ± S.D.^a^	20.1 ± 7.3	23.6 ± 8.8	Current-smoker	42(48.3)	Adenocarcinoma	42(57.5)
	Median	20.0	23.0	Ex-smoker	15(17.2)	Squamous cell ca	22(30.1)
	Min^b^-Max^c^	2.5-41.0	7.0-61.0	Never smoker	30(34.5)	Others	9(12.3)

^a^S.D. is the abbreviation of standard deviation; ^b^Min is minimal value; ^c^Max is maximal value

**Table 13:** The durations of exposure and latency, smoking status and pathologic types of compensated lung cancers by the kinds of main carcinogens in Occupational lung diseases institute, Korea (2004-2011)

Exposed substances (No. of lung cancer)		Exposure duration (years)	Latent duration (years)	Smoking status	No. (%)	Type of pathology	No. (%)
Asbestos (30)	Mean ± S.D.^a^	22.7 ± 8.4	26.5 ± 7.6	Current-smoker	18(60)	Adenocarcinoma	12(40)
	Median	22	24.5	Ex-smoker	5(16.7)	Squamous cell ca	12(40)
	Min^b^-Max^c^	10-50	16-50	Never smoker	6(20)	Others	6(20)
Crystalline silica (2)	Mean ± S.D.^a^	22 ± 11.3	25.5 ± 13.4	Current-smoker	2(100)	Adenocarcinoma	1(50)
	Median	22	25.5	Ex-smoker	0(0)	Squamous cell ca	1(50)
	Min^b^-Max^c^	14-30	16-35	Never smoker	0(0)	Others	0(0)
Cr6+ & welding fume (11)	Mean ± S.D.^a^	22.3 ± 8.8	27.1 ± 7.0	Current-smoker	8(72.7)	Adenocarcinoma	5(45.5)
	Median	22	29	Ex-smoker	1(9.1)	Squamous cell ca	6(54.6)
	Min^b^-Max^c^	10-34	16-35	Never smoker	1(9.1)	Others	0(0)
Others (10)	Mean ± S.D.^a^	21.3 ± 3.9	22.7 ± 4.3	Current-smoker	5(50)	Adenocarcinoma	4(40)
	Median	22	22.5	Ex-smoker	2(20)	Squamous cell ca	3(30)
	Min^b^-Max^c^	16-28	16-30	Never smoker	3(30)	Others	3(30)
Single exposure (11)	Mean ± S.D.^a^	23.2 ± 11.0	30 ± 8.7	Current-smoker	7(63.6)	Adenocarcinoma	5(45.5)
	Median	20	30	Ex-smoker	2(18.2)	Squamous cell ca	3(27.3)
	Min^b^-Max^c^	10-50	19-50	Never smoker	2(18.2)	Others	3(27.3)
Co-exposure (20)	Mean ± S.D.^a^	22.1 ± 6.8	24.8 ± 6.2	Current-smoker	12(63.2)	Adenocarcinoma	8(40)
	Median	22	24	Ex-smoker	3(15.8)	Squamous cell ca	9(45)
	Min^b^-Max^c^	10-34	16-35	Never smoker	4(21.1)	Others	3(15)

^a^S.D. is the abbreviation of standard deviation; ^b^Min is minimal value; ^c^Max is maximal value

Cases for which asbestos was surveyed as a key carcinogen or a secondary carcinogen were selected and analyzed as follows. When classified by industry and occupation, the manufacturing industry in the high-level industry classification accounted for the highest number with 70 cases, including construction (31 cases), followed by transportation (23 cases). Industries and occupations with 2 or more asbestos-related lung cancer patients included maintenance and spinning (textile) in the other fiber (asbestos) spinning (or textile) industry; construction material manufacturing in the asbestos, mineral woolen and other similar product manufacturing industry; machine system installation and repair in the petroleum refining industry; machine system installation and repair in the basic iron and steel manufacturing industry; welding in the structural metal parts manufacturing industry; brake lining assembly in the motor vehicle assembly industry; brake lining manufacturing in the motor vehicle parts manufacturing industry; welding and ship machinery in the shipbuilding industry; ship assembly in the ship parts manufacturing industry; welding, insulation and plumbing in the plant construction industry; scaffolding in the scaffolding industry; insulation and welding in the cooling and heating and plumbing related industry; driver, attendant and maintenance in the railroad train, underground train transportation industry; bus driving, repair and maintenance in the city bus transportation industry; and boiler operation in the real estate management industry. Boiler-related occupations (operation and maintenance) were noted throughout all industries (Table 14).

**Table 14:** The Classification of industry and job and exposed carcinogens in compensated asbestos-related lung cancers in Korea (1994-2011)

Industry classification (No of cases)	Industry classification	Job classification (No of cases)	No of cases	Main carcinogen	Minor carcinogen
Mining and quarrying (1)	Mining of non-ferrous (tungsten) metals	Repairer & welder	1	Asbestos	Welding fume
Manufacturing (70)	Bean curd and similar products	Boiler operators	1	Asbestos	PAH^b^
	Spinning (or weaving) of other textile fibers (asbestos)	Maintenance & repairer (4)	10	Asbestos	
		Spinning (or weaving) worker (6)			
	Other printing	Paper arrangement worker	1	Asbestos	
	Petroleum refineries	Machinery equipment fitters and repairers	2	Asbestos	
	Synthetic fibers	Spinning (or weaving) (1)	2	Asbestos	PAH^b^ (1)
		Boiler operator (1)			
	Pottery and ceramic household or ornamental ware	Pottery making (burning)	1	Silica	Asbestos
	Asbestos, mineral wools and other similar products	Gasket & sheet manufacture (mixing) (1)	8	Asbestos	
		Construction material manufacturers (slate, heat insulator, et al) (6)			
		Machinery equipment fitters and repairers (1)			
	Basic iron	Metal furnace operators	1	Asbestos	
	Basic steel	Electrical furnace operator(1)	4	Cr^6+^	Asbestos
		Rolling mii operator (1)		Asbestos	Cr^6+^,Nickel
		Crane operator(1)		Cr^6+^	Asbestos, Nickel
		Machinery equipment fitters and repairers (welding)(1)		Asbestos	
	Rolled, drawn and folded products of aluminum	Machinery equipment fitters and repairers (plumber)	1	Asbestos	
	Gray and malleable iron foundries / Steel foundries	Foundry workers: melting, molding, core making, fettling	1	Silica	asbestos
	Metal structural components	Welding	4	Asbestos, Cr^6+^	Welding fume
	Central heating boilers and radiators	Boiler maker	1	Asbestos	Silica, F, Metal
	Weapons and ammunition	Plumbing & welding	1	Asbestos	Welding fume
	Heat treatment of metals	Heat treatment & welding	1	PAH^b^	Cr^6+^, Asbestos, FA
	General hardware	Die and mold makers	1	Asbestos	
	Hand-operated kitchen appliances and metal ware	Fitting & welding	1	Asbestos	Welding fume
	Air conditioning and control machines	Machine manufacturer	1	Asbestos	
	Motor vehicles	Automobile parts (brake lining) assemblers	3	Asbestos(3)	
	Motor vehicles for the transport of goods and special purpose	Building and machine repairers	1	Asbestos	
	Other parts and accessories for motor vehicles n. e. c.	Automobile parts (brake lining or gasket) manufacturer (5)	6	Asbestos (6)	F
		Foundry works (1)		Silica	
	Building of steel ships	Welding (7)	13	Asbestos(7), Coaltar(1)	Welding fume(6), Cr^6+^,Asbestos(1)
		Painting (1)		Asbestos (1)	
		Plumbing (1)		Asbestos(1)	
		Insulation (1)		Asbestos(1)	
		Electrical equipment engineers (1)		Asbestos(2)	
		Ship mechanics (2)			Silica(1), Cr^6+^&PAH^b^
					F(2)
	Sections for ships	Ship assemblers	4	Asbestos	
		Ship engineers and repairers		Asbestos, Cr^6+^	
		Painters			
	Upholstered seats for transport vehicles	Drying oven fitters	1	Asbestos	
Sewage, waste management, materials recovery and remediation activities (1)	Construction and demolition waste collection	Waste collection and transportation	1	DEE^a^	Asbestos
construction(31)	Construction of highways, streets and roads	Road repairers (1)	5	Asbestos(1)	
	Installation of environmental hygiene treatment appliances	Construction waste transport workers	1	Asbestos	DEE^a^
	Construction of industrial plants	Welding(5)	13	Welding fume (4), Asbestos (1)	Asbestos(4), Welding fume(1)
		Insulation (5)		Asbestos(5)	
		Pipe making (2)		Asbestos(2)	
	Wrecking and demolition of buildings and other structures (ships)	Ship repairers and wreckers	1	Asbestos	
	Scaffolding and frame works	Scaffolders (3)	3	Asbestos (3)	
	Heating, air conditioning and plumbing related works	Insulation (4)	7	Asbestos (4)	Welding fume(1)
		Boiler fitters and mechanics (1)		Asbestos (1)	
		Welding (2)		Asbestos(1), Welding fume(1)	Asbestos(1)
	Other building completion n.e.c.	Wrecking & interior	1	Asbestos	Welding fume
Transportation (23)	Interurban rail transportation	Railway signalmen and repairers	1	Asbestos	Radon
	Commuter rail systems	Electric train drivers (3)	16	Asbestos(3)	Radon(3)
		Electric train attendants (6)		Asbestos(6)	Radon(6)
		Repairers and maintenance (7)		Asbestos(7)	Welding fume & Radon & DEE^a^ (4)
					Radon&DEE^a^ (1)
					Radon(1), Welding fume(1)
	Urban bus passenger transport	Bus driver & repairer (2)	6	DEE^a^ (1), Asbestos(1)	Asbestos(1),DEE^a^ (1)
		Bus repairer & maintenance (4)		Asbestos(3), DEE^a^ (1)	DEE^a^ (1), Asbestos(1)

^a^DEE is the abbreviation of diesel engine exhaust; ^b^PAH is polyaromatic hydrocarbons

When these industries and occupations were analyzed in light of work environment monitoring results based on the criterion of 4 fiber-year with the relative risk of lung cancer of 2, as proposed by Gustavsson et al. [5], all occupations satisfied the criterion of 4 fiber-year in 5 years, except for boiler-related occupations. Furthermore, these industries and occupations met the definition of work involving asbestos exposure according to standards for industrial accident compensation in the approval standards of Japan.

## Conclusion

### Proposal of new approval standards of occupational cancers due to asbestos exposure

Since announced, the Helsinki Criteria served as the approval standards or guidelines for asbestos-related lung disease in many countries. However, there were numerous discussions on the criteria and approval standards have been revised in a number of countries. As the post-Helsinki discussion in Korea, this study reviewed the use of CT in recognition of lung cancer due to asbestos, criteria of asbestosis on CT, asbestos exposure concentrations in recognition of lung cancer due to asbestos, relationship between cigarette smoke and asbestos in causing lung cancer, latent duration between asbestos exposure and lung cancer, and relationship between larynx cancer and lung cancer.

As described previously, the current approval standards of asbestos-related diseases in Korea have just copied Japanese approval standards of decades ago, and new standards enacted in July 2013 are still unspecific and vague. Therefore, this study proposed new approval standards of occupational cancers due to asbestos, based on post-Helsinki discussions, work environment monitoring data in Korea, and analysis of lung cancer cases recognized as an industrial accident.

① In recognizing an asbestos-induced lung cancer, diagnosis of asbestosis should be based on CT.

Several studies have reported a high incidence of lung cancers even without asbestosis on simple chest X-ray. Even when asbestosis was not found with chest radiography, the odd ratio for lung cancer increased with a longer duration of cumulative asbestos exposure. was an additional risk factor and exhibited a weaker dose-response relationship than the cumulative exposure duration. HRCT is already in use in a number of countries in diagnosing lung diseases due to asbestos. CT was found to be highly useful in terms of sensitivity, specificity and positive predictive value. Subpleural dotlike opacities and subpleural curvilinear opacities on HRCT are noted for early stage asbestosis, and over the course of disease, intralobular interstitial thickening or intralobular lines and interlobular septal thickening are observed.

② Industries and occupations with high exposure to asbestos in Korea should be taken into account.

When industries and occupations with 2 or more asbestos-related lung cancer patients were analyzed in work environment monitoring results based on the criterion of 4 fiber-year with the relative risk of lung cancer of 2, as proposed by Gustavsson et al. [5], all occupations satisfied the criterion of 4 fiber-year in 5 years, except for boiler-related occupations. Furthermore, these industries and occupations met the definition of work involving asbestos exposure according to standards for industrial accident compensation in the approval standards of Japan.

③ An expert’s determination is warranted in case of a worker who has been concurrently exposed to other carcinogens, even if the duration after asbestos exposure is less than 10 years.

In most countries, approval standards of asbestos-related diseases require that at least 10 years should have passed since asbestos exposure. In most epidemiological studies, asbestos-related cancers develop 10 years after exposure. However, according to KOSHA and Occupational Lung Diseases Institute between 1994 and 2011, lung cancer cases recognized as an industrial accident in Korea involved exposure to multiple carcinogens, with 50 % or more in case of the KOSHA data and approximately 65 % for the Occupational Lung Diseases Institute data. As there have been few studies of the risk of lung cancer due to concurrent exposure to asbestos and other carcinogens, it is warranted to seek an expert’s judgment in case of multiple exposures.

④ Determination of a larynx cancer due to asbestos exposure has the same approval standards with an asbestos-induced lung cancer. However, for an ovarian cancer, an expert’s judgment is necessary even if asbestosis, pleural plaque, pleural thickening and high concentration asbestos exposure are confirmed.

Larynx cancer has a dose-response relationship with asbestos exposure, as lung cancer However, in case of an ovarian cancer, there is no available domestic epidemiological survey for asbestos-related ovarian cancer and no cases have been claimed or recognized so far. While some overseas data claim evidence of the association between asbestos and ovarian cancer, only a few epidemiological studies [35, 4] have been conducted. Therefore, an expert’s judgment is warranted for recognition in case an asbestos-related ovarian cancer is submitted for application of an industrial accident.

⑤ Cigarette smoking status or the extent should not affect determination of an occupational cancer caused by asbestos as smoking and asbestos have a synergistic effect in causing a lung cancer and they are involved in carcinogenesis in a complicated manner.
